# Supplementary material for: Estetrol Combined to Progestogen for Menopause or Contraception Indication Is Neutral on Breast Cancer
Source: Cancers (Basel). 2021 May 20;13(10):2486. doi: 10.3390/cancers13102486 (PMC8160902; doi:10.3390/cancers13102486)
Supplement: Supplementary file 1 [file cancers-13-02486-s001.zip › cancers-1178167_supplementary proof/cancers-1178167_supplementary proof revised.pdf]

Supplemental Materials and Methods

Human treatment modeling in mice: dose delineation

In order to model human treatments, it is essential to administer steroids to mice in a pattern that closely mimics steroid exposure in women (Table S1). E2 has been chosen as a reference treatment, since it is recognized as the most widely used for MHT [1]. The steroid doses administered to mice were defined to match the human therapeutic dose of E2 and P4 recommend by the FDA and EMA for MHT, with E2 (0.5-2 mg/day) and P4 (100 or 200 mg/day) [31-33]. For E4 and DRSP the dose choice was based on clinical studies [8,12,16-18]. Chronic oral administration of E2 (2 mg/day) tablets results in a steady-state of 0.070 ng/ml E2 plasma concentration [33] that was mimicked in mice by a slow-releasing E2 implant (0.08 mg/kg/day). Two doses (1.25 and 4.25 mg/kg/day) of P4 were administered by slow-releasing implants to mice to cover the range of P4 doses recommended for the prevention of endometrial proliferation in association with estrogen therapy (100 mg/day or 1.66 mg/kg/day to 200 mg/day or 3.33 mg/kg/day). Since E4 has a different half-life in humans (28-32h) and in mice (2h) [10,12], the pharmacokinetic profile of E4 differs between women and mice. Thus, to mimic the steady-state obtained by once-a-day oral treatment in women [38], we continuously administered E4 with Alzet® osmotic pumps to mice (Figure S1,A-B). E4 was used at 0.3 mg/kg/day in mice to mimic the E4 therapeutic dose (15 mg/day) for COC or MHT in women [8,17,18] since E4 plasma concentration in mice for a dose of 0.3 mg/kg/day fell into the range of plasmatic concentration in women reported in a clinical study [38] (Table S1). In addition, a supratherapeutic dose of E4 (3 mg/kg/day), corresponding to 10-fold the therapeutic dose, was also evaluated (Figure S1,B). DRSP was combined to E4 (0.3 mg/kg/day E4 / 0.06 mg/kg/day DRSP) within the same ratio as in women (15 mg E4 / 3 mg DRSP) [17,18].

Cell proliferation

MCF7 and T47D cells were seeded in 96 well-plates (10^3 cells/well) in DMEM supplemented with 10% fetal bovine serum (FBS) for 24h. Then, cells were cultured for 24h in red phenol-free medium (Gibco Invitrogen Corporation, Paisley, UK) supplemented with 10% heat-inactivated and dextran-coated charcoal treated FBS (FBS-cs, Lonza, Basel, Switzerland). Estrogenic treatments (E2 10^{-9} M or E4 10^{-10}M or 10^{-7}M) or vehicle (ethanol 0.01%) were initiated and proliferation was assessed after 24, 48 and 72h of treatment using the Cyquant™ Cell Proliferation Assay Kit (Life Technologies, Invitrogen, Thermo Fisher Scientific, Waltham, MA, USA). The experiments were repeated 2-3 times. One representative experiment in presented for both MCF7 and T47D cell lines. For MCF7 cells and T47D, 9-15 and 7-9 independent replicates were evaluated respectively.

Lung metastasis quantification

To evaluate lung metastatis dissemination in the MMTV-PyMT model, paraffin-embedded lung sections (5µm) were stained with hematoxylin and eosin (H/E). Four slides separated by 50 µm were collected from each mouse. Numeric images were obtained with a NanoZoomer 2.0-digital slide scanner (Hamamatsu Photonics, Hamamatsu City, Japan).

On each slide, lung metastasis number, lung metastasis area and total lung area were measured by computer assisted image analysis using Matlab software (MathWorks, Inc., Natick, MA M01760, USA). The metastasis number was calculated as the ratio of lung metastasis number/total lung area, corresponding to the lung metastasis number density ($1/mm^2$). The metastasis mean size was calculated from the measurement of the area (mm^2) of each metastatic focus. The total metastasis area corresponds to the percentage of lung area occupied by metastasis, which was calculated as the ratio of the sum of all metastasis foci area/total lung area. For one mouse, each parameter was calculated as the mean of the 4 values measured from the 4 slides of the same lung.

Immunohistochemical staining for Ki67, ERα, pS118-ERα and PR

Uteri and tumors were collected at sacrifice and paraffin embedded. Immunolabeling was carried out on serial 5µm sections using anti-Ki67 (#Ab16667, Dako, Glostrup, Denmark), anti-ERα (SP1, #790-4325, Ventana, Roche Diagnostics GmbH, Mannheim, Germany), anti-phospho S118-ERα (16J4, #2511, Cell signaling Technology, Danvers, MA 01923, USA), anti-PR (1E2, #790-4296, Ventana, Roche Diagnostics GmbH, Mannheim, Germany) antibodies, followed with the appropriate secondary anti-rabbit or anti-mouse Envision system -HRP antibodies (#K4003 and #K4001, Dako, Glostrup, Denmark). Immunolabeling was revealed with DAB (3,3'-diaminobenzidine) and Chromogen System (Dako, Glostrup, Denmark). Tissues were counterstained with hematoxylin and mounted with Entellan @new (Millipore, Merck KGaA, Darmstadt, Germany). Numeric images were acquired with a NanoZoomer 2.0-digital slide scanner (Hamamatsu Photonics, Hamamatsu City, Japan). Staining and quantifications were performed on 7-9 independent MCF7 tumors and on 10-20 independent PDX tumors. Image analysis quantifying tumor staining density was performed with Matlab software (MathWorks, Inc, Natick, MA M01760, USA) as previously described [36]. Briefly, the density of staining was calculated as the ratio between the tumor area and the area occupied by positive PR, pS118-ERα, ERα or Ki67 staining, then it was normalized to the number of cells. Luminal epithelium height of uteri was measured with a NanoZoomer Digital Pathology viewer (Hamamatsu Photonics, Hamamatsu City, Japan).

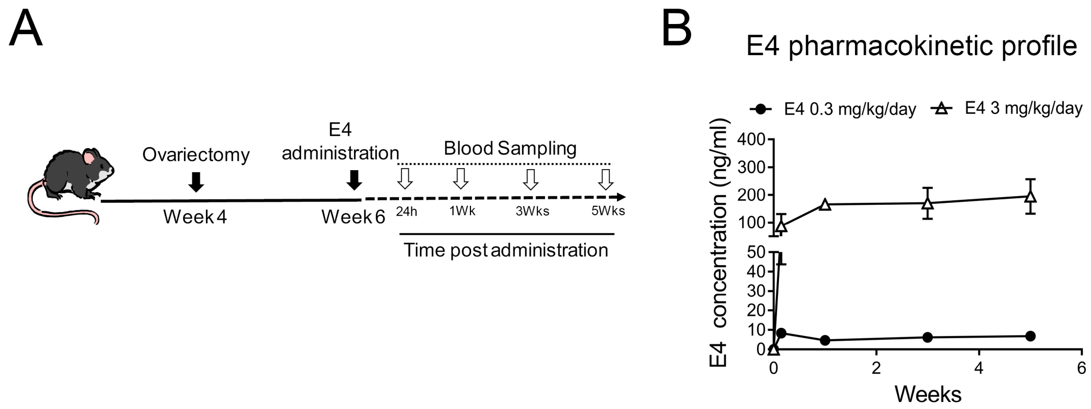

Figure S1 : E4 pharmacokinetics during continuous delivery in mice
(**A**) Schematic representation of mouse treatment and blood sampling for the pharmacokinetic study. Ovariectomized MMTV-PyMT mice were treated subcutaneously with osmotic Alzet Pumps® releasing E4 at 0.3 or 3 mg/kg/day for 5 weeks. Blood sampling was performed after 24h, 1 week, 3 weeks and 5 weeks of treatment. (**B**) E4 blood concentration over time. Results are expressed as mean ± SEM ; n=6 blood samples/condition.

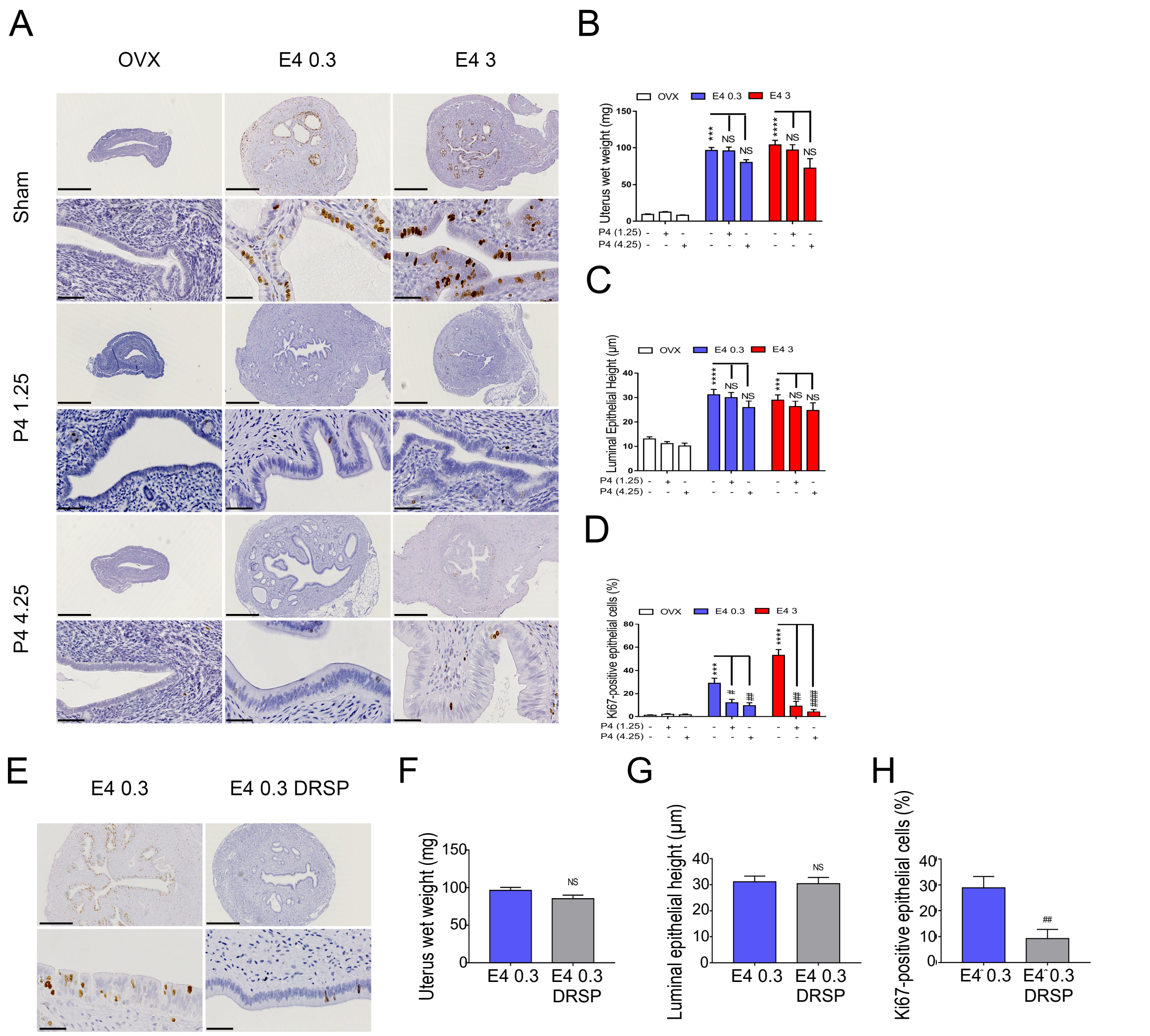

Figure S2: Uterotrophic effect of E4 and P4 in MCF7 cell xenograft mouse model

(**A**) Representative Ki67 immunostainings on uterus harvested from MCF7-grafted Swiss Nu/Nu mice untreated (OVX) or treated with E2, E4 (0.3 or 3 mg/kg/day) combined with or without P4 (1.25 or 4.25 mg/kg/day); scale bar=500µm, zoom scale bar=50µm. Quantification of (**B**) uterine wet weight, (**C**) luminal epithelial height and (**D**) epithelial cell proliferation (Ki67-positive staining). (**E**) Representative Ki67 immunostainings on uterus harvested from MCF7-grafted treated with E4 (0.3 mg/kg/day) with or without DRSP (0.06 mg/kg/day); scale bar=500µm, zoom scale bar=50µm. Quantification of (**F**) uterine wet weight, (**G**) luminal epithelial height and (**H**) epithelial cell proliferation (Ki67-positive staining). Kruskal-Wallis analysis followed by Dunn's post-tests or Mann-Whitney analysis, n=8-15 mice/condition. NS: not statistically significant; * or #: p<0.05; ** or ##: p <0.01; *** or ###: p <0.001 and **** or ####: p <0.0001. * *versus* OVX, # *versus* corresponding sham.

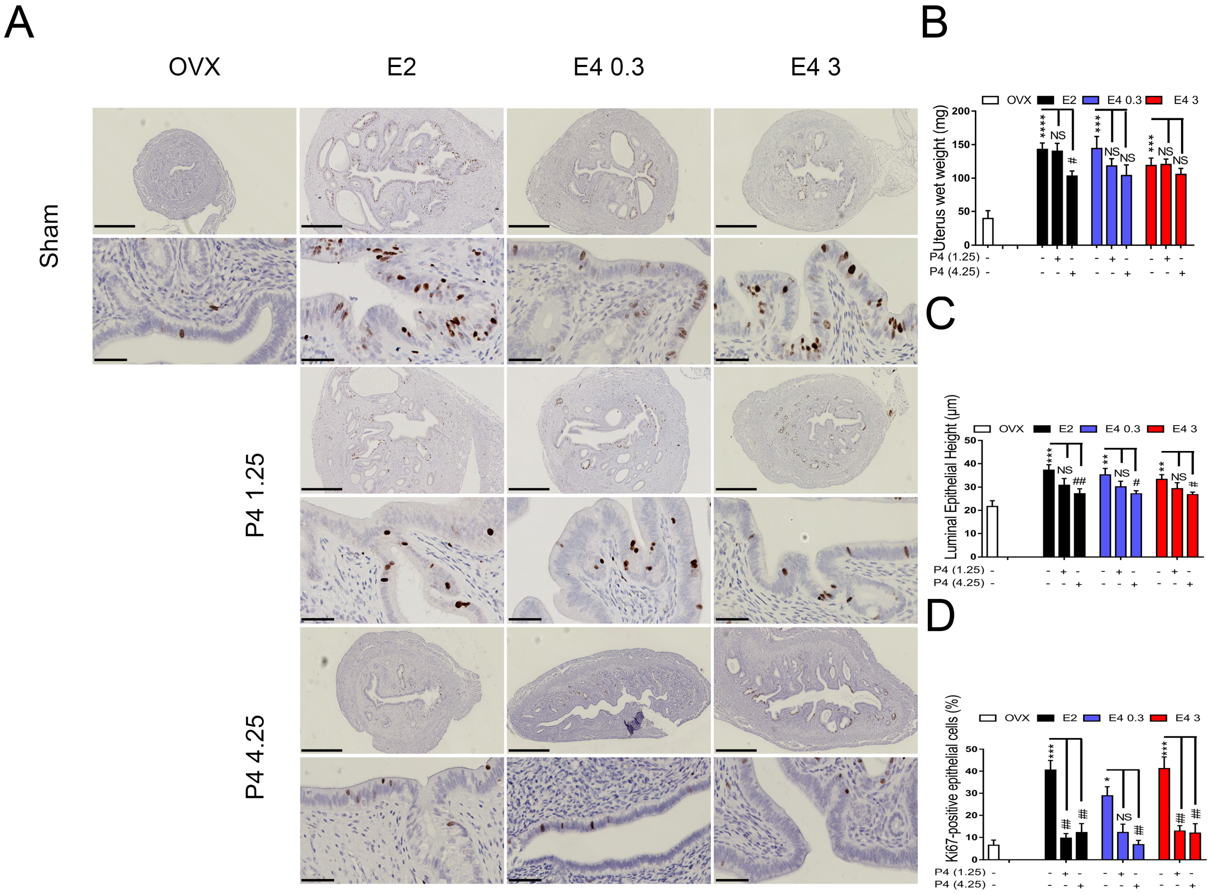

Figure S3 : Uterotrophic effect of E4 and P4 in hormone-dependent PDX mouse model
(**A**) Representative Ki67 immunostainings on uterus harvested from PDX-grafted mice untreated (OVX) or treated with E2, E4 (0.3 or 3 mg/kg/day) combined with or without P4 (1.25 or 4.25 mg/kg/day); scale bar=500µm, zoom scale bar=50µm. Quantification of (**B**) uterine wet weight, (**C**) luminal epithelial height and (**D**) epithelial cell proliferation (Ki67-positive staining). Kruskal-Wallis analysis followed by Dunn's post-tests, n=7-14 mice/condition. NS: not statistically significant; * or #: $p<0.05$; ** or ##: $p<0.01$; *** or ###: $p<0.001$ and **** or ####: $p<0.0001$. * *versus* OVX, # *versus* corresponding sham.

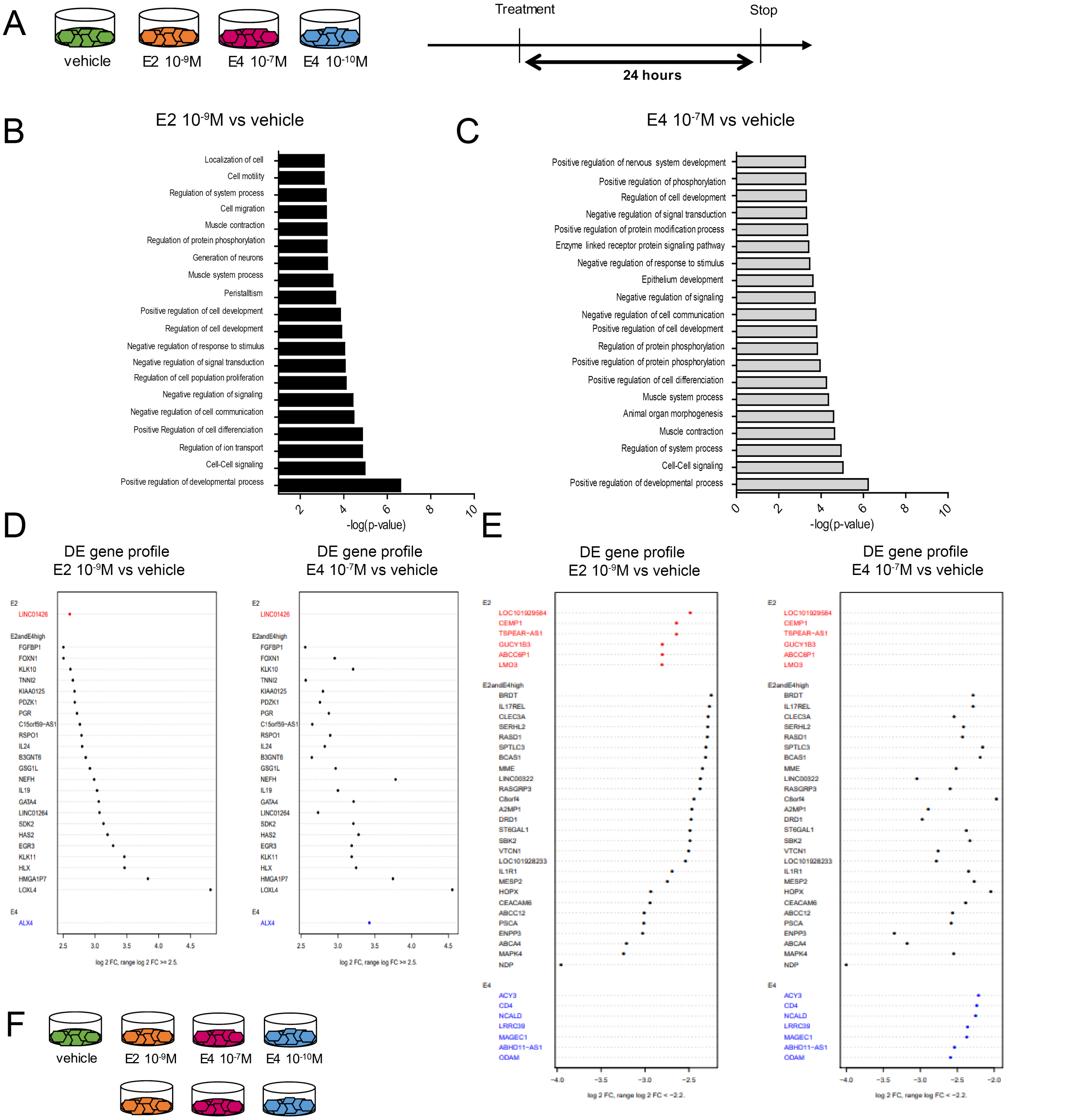

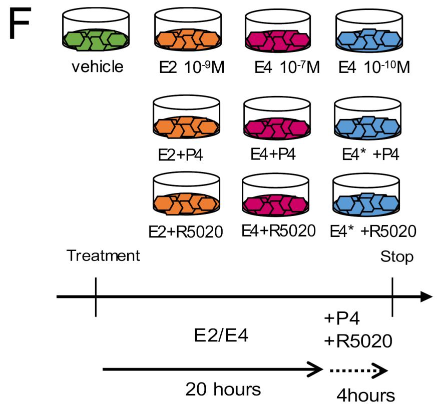

Figure S4: Transcriptomic analysis of E2 and E4 treatments
(**A**) RNAseq schematic estrogen-only treatments performed on MCF7 cells for 24h. (**B,C**) Ingenuity® Pathways analysis revealing functional activity of genes regulated by (**B**) E2 (10^{-9}M) or by (**C**) E4 (10^{-7}M). (**D**) Genes upregulated by E2 (10^{-9}M) and E4 (10^{-7}M) with a Log2 fold change ranging from 2.5 to 4.5. (**E**) Genes downregulated by E2 (10^{-9}M) and E4 (10^{-7}M) with a Log2 fold change ranging from -2 to -4. Genes regulated by either E2 or E4 are presented in red or blue, respectively; genes commonly regulated are in black. (**F**) RNAseq schematic estrogen-progestogen treatments performed on MCF7 cells for 24h; E4*=10^{-10}M E4, E4=10^{-7}M E4; P4 (10^{-7}M), R5020 (10^{-8}M).

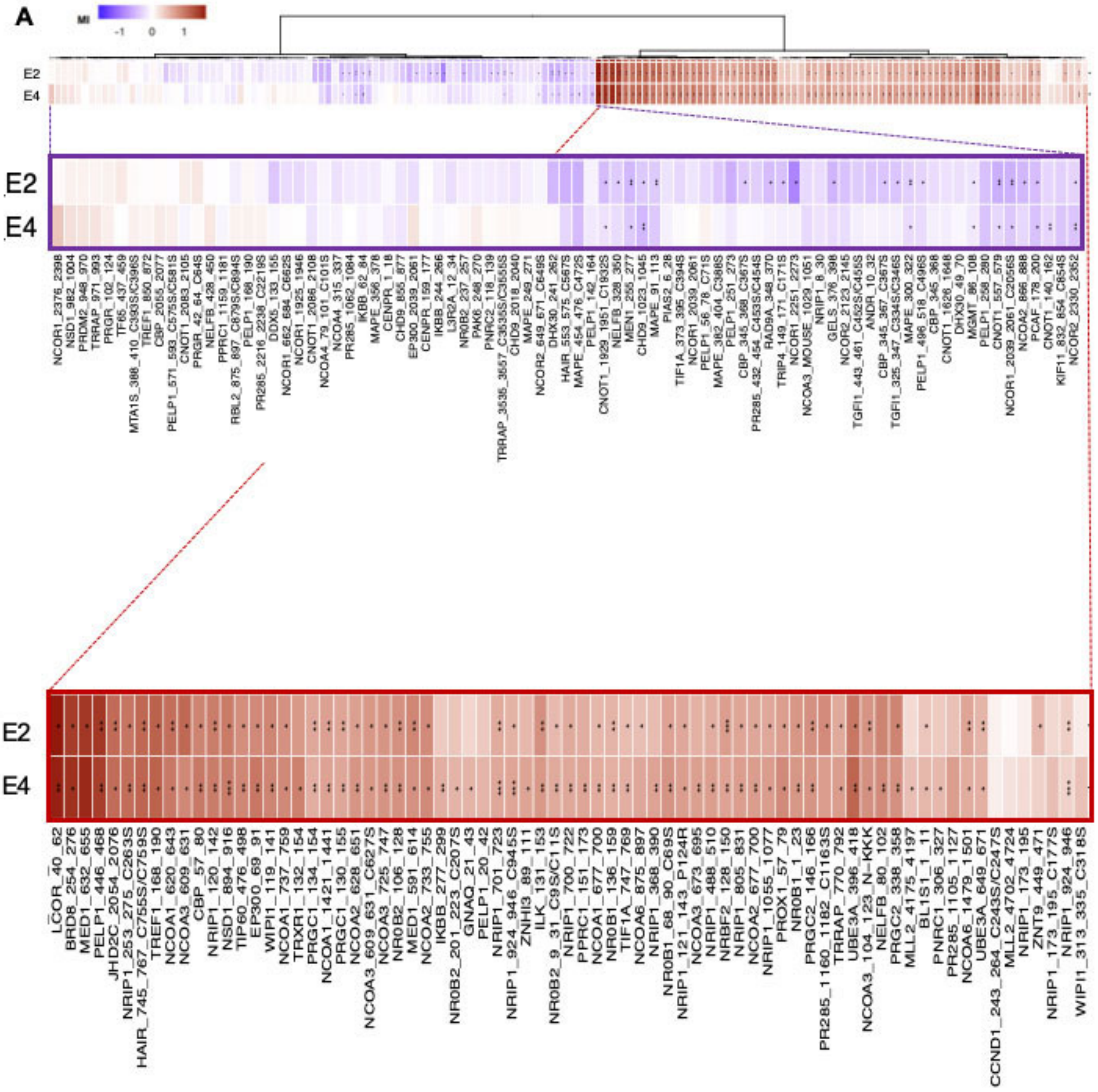

Figure S5: MARCoNI assay

Heatmap of interactions between ERα and co-regulators induced by E2 or E4 and represented as the modulation index (MI), with zoom outs from the two main clusters. MI is expressed as the log of fold-changes relative to vehicle.

Table S1: Steroid dose equivalence between human and mouse

Species	Estrogen	Doses	Mean plasmatic concentration (ng/ml)	Progestogen	Doses
Human	E2	2 mg/day	0.070 (27)	P4	100-200 mg/day (25-27)<br>(1.66-3.33 mg/kg/day)
	E4	2-40 mg/day	1.29 – 82.73 (36)	DRSP	3 mg/kg (17, 18)<br>(0.05 mg/kg/day)
Mice	E2	0.08 mg/kg/day	0.116 ± 0.016	P4	1.25 mg/kg/day
	E4	0.3 mg/kg/day	5.19 ± 1.42	P4	4.25 mg/kg/day
	E4	3 mg/kg/day	124 ± 36	DRSP	0.06 mg/kg/day

Dose equivalence and plasmatic concentrations of steroids were analyzed and compared between human and mice. Human data were obtained from the literature. Mice received chronic subcutaneous treatment by mini osmotic Alzet pumps (for E4 and DRSP) or matrix pellets (for E2 and P4). Results are expressed as mean ± SEM, n= 6.

Table S2: Primer sequences for RT-qPCR	
**Gene**	**Sequences**
**PGR**	Forward 5′-GGC-ATG-GTC-CTT-GGA-GGT-3′<br>Reverse 5′-CCA-CTG-GCT-GTG-GGA-GAG-3′
**TBP**	Forward 5′-GAC-TCC-CAT-GAC-CCC-CAT-3′<br>Reverse 5′-CAA-CCA-AGA-TTC-ACT-GTG-GAT-AC-3′
**GAPDH**	Forward 5′-TGC-CGT-CTA-GAA-AAA-CCT-GCC-AAA-3′<br>Reverse 5′-CTC-TCT-TCC-TCT-TGT-GCT-CTT-GCT-3′
