# Supplementary material for: Estetrol Combined to Progestogen for Menopause or Contraception Indication Is Neutral on Breast Cancer
Source: Cancers (Basel). 2021 May 20;13(10):2486. doi: 10.3390/cancers13102486 (PMC8160902; doi:10.3390/cancers13102486)
Supplement: Supplementary file 1 [file cancers-13-02486-s001.zip › cancers-1178167_supplementary proof/Suppl Material_Fig 5 Panel A_WB whole blot 2.pdf]

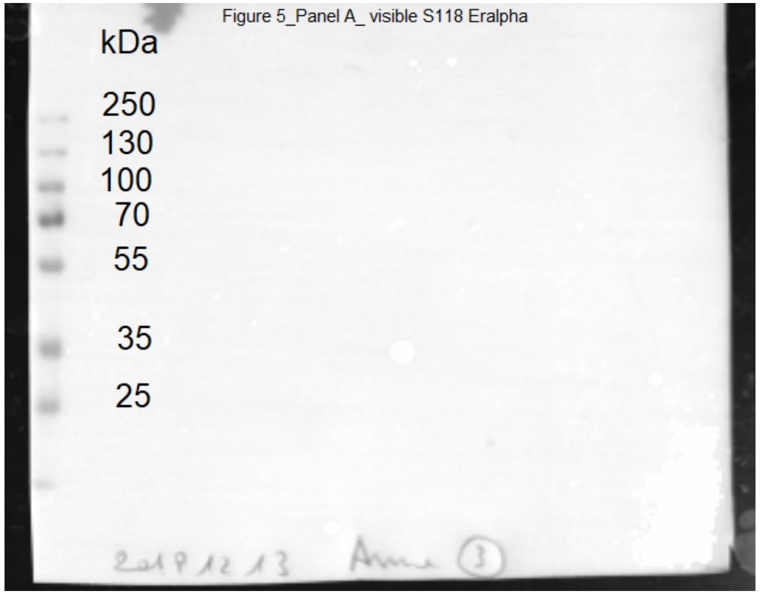

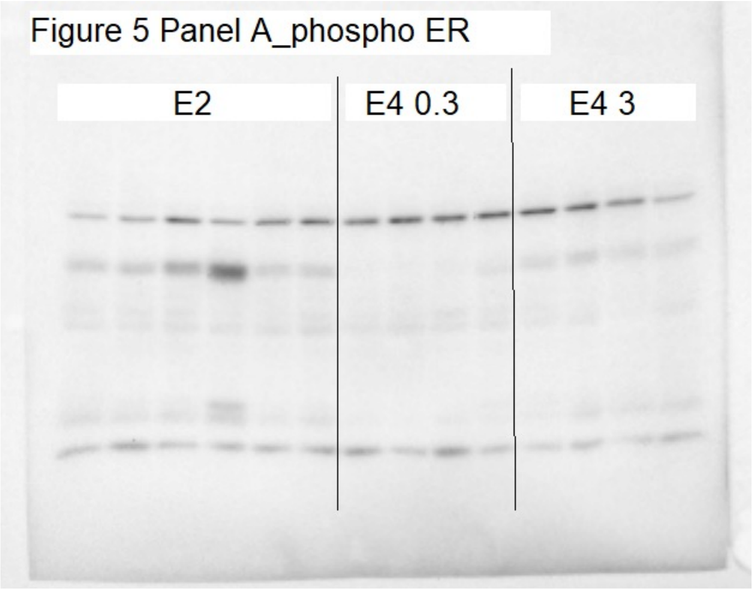

pS118-ERalpha 66kDa

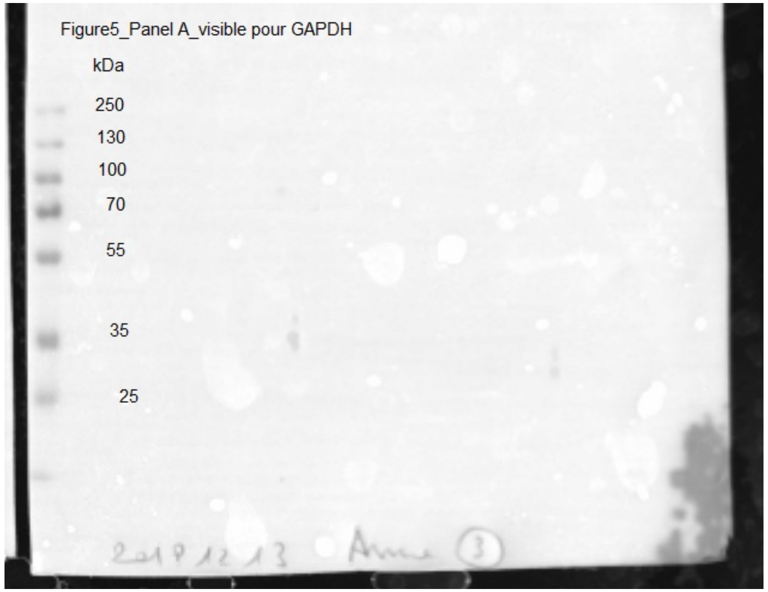
Figure5_Panel A_visible pour GAPDH
kDa
250
130
100
70
55
35
25

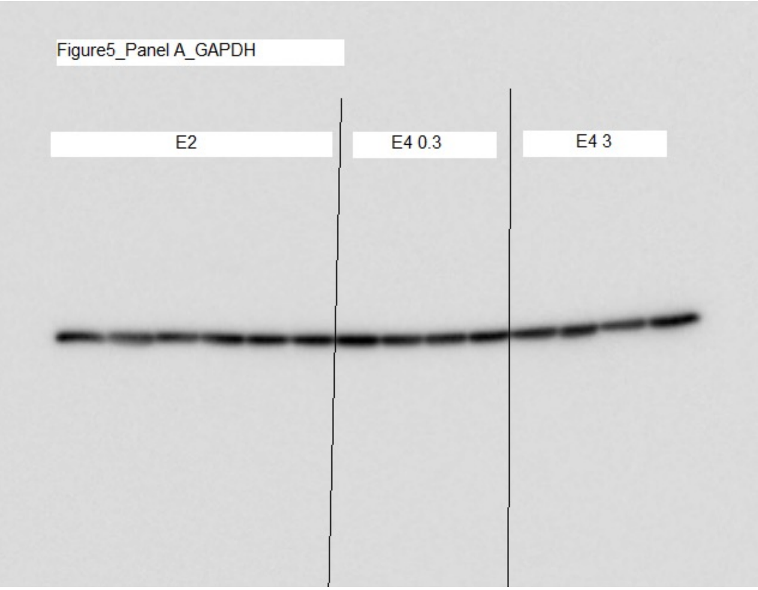
Figure5_Panel A_GAPDH
E2
E4 0.3
E4 3

GAPDH 37kDa

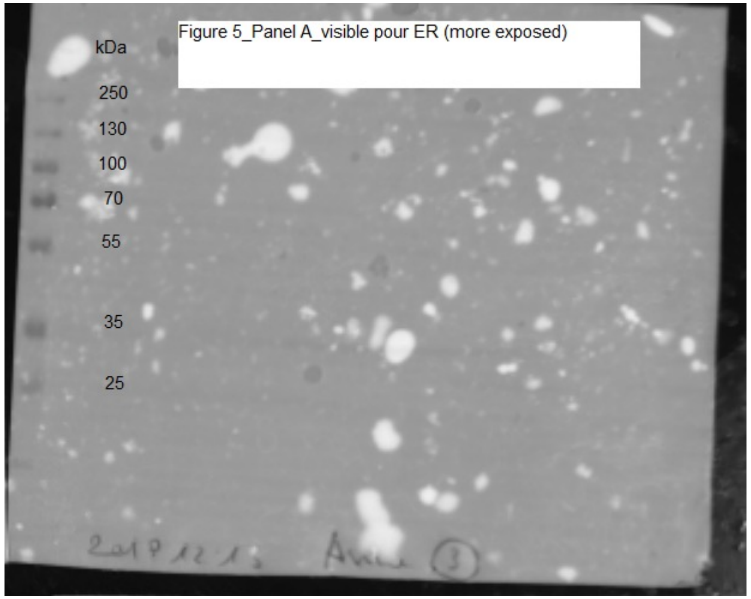

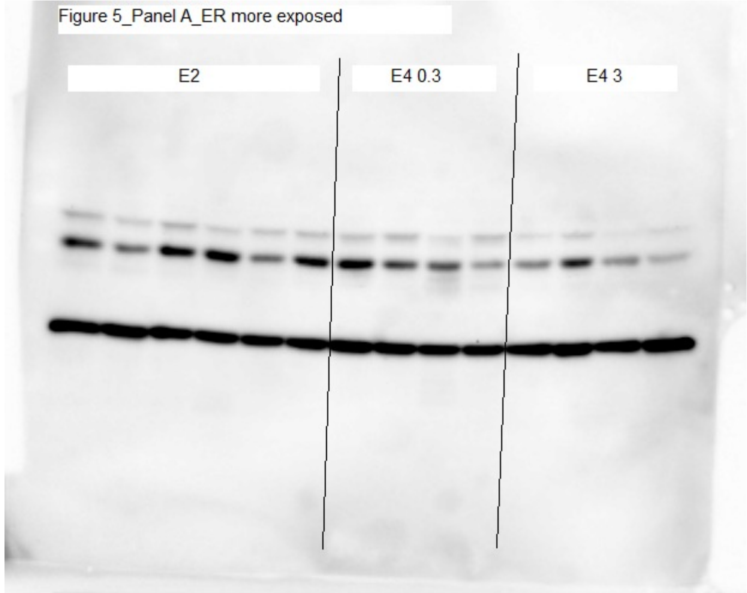

ERalpha 66kDa, 54kDa

Immunostainings have been made successively on the same membrane, using stripping between two immunostainings.

Figure 5_Panel A_PR

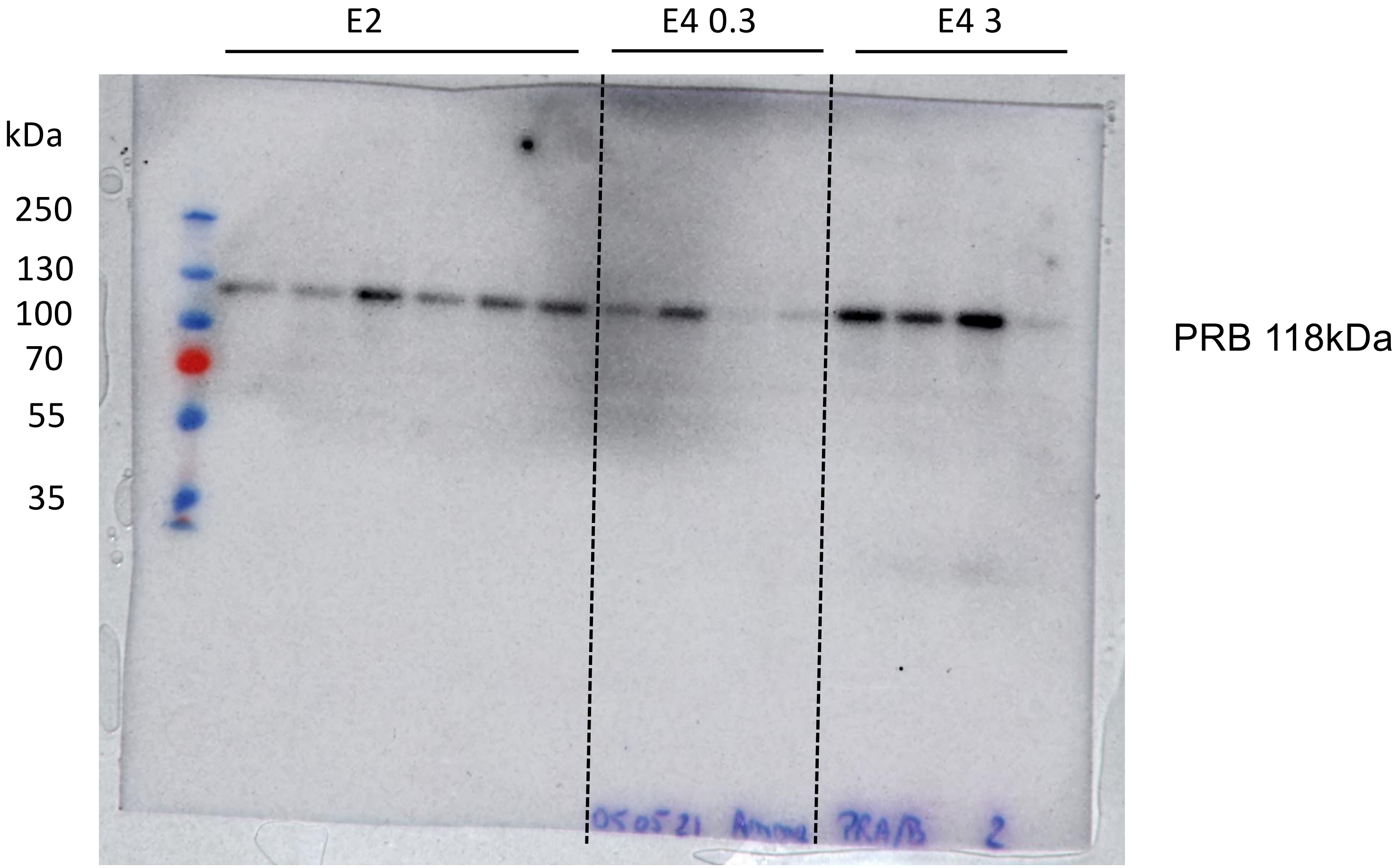

MCF7 Tumors Western blot quantification

Figure 5 Panel A

PR/GAPDH Quantification

E2	E4 0.3	E4 3
7,05149382	7,67260386	6,51067051
7,32381518	6,08351359	6,79303214
7,4091081	6,17318763	7,47170443
6,9707311	7,07740892	6,90225236
6,73759318	7,8083337	8,72834594
8,6289407	4,35667894	8,05131712
8,82229658	4,99482605	7,68069838
7,72233509	5,77207014	6,23389095
6,95305499		

MCF7 Tumors Western blot quantification

Figure 5 Panel A

S118Eralpha/ERalpha Quantification

E2	E4 0.3	E4 3
2,94729097	2,67091448	1,81431439
3,33240242	3,62002427	2,73804205
1,93943406	3,10962931	4,11197765
2,04838593	2,06068449	2,1270495
9,00806468	3,79974538	8,44331019
8,77690365	3,7567859	6,74745546
6,87815235	2,010441	8,48838761
4,45484731	4,58418287	7,04203997
5,38126526		

n= 8-9 independant replicats
All the samples were run in two gels.
Each sample was run two times independently.
