# Supplementary material for: Estetrol Combined to Progestogen for Menopause or Contraception Indication Is Neutral on Breast Cancer
Source: Cancers (Basel). 2021 May 20;13(10):2486. doi: 10.3390/cancers13102486 (PMC8160902; doi:10.3390/cancers13102486)
Supplement: Supplementary file 1 [file cancers-13-02486-s001.zip › cancers-1178167_supplementary proof/Suppl Material_Fig 4 Panel C_WB whole blot.pdf]

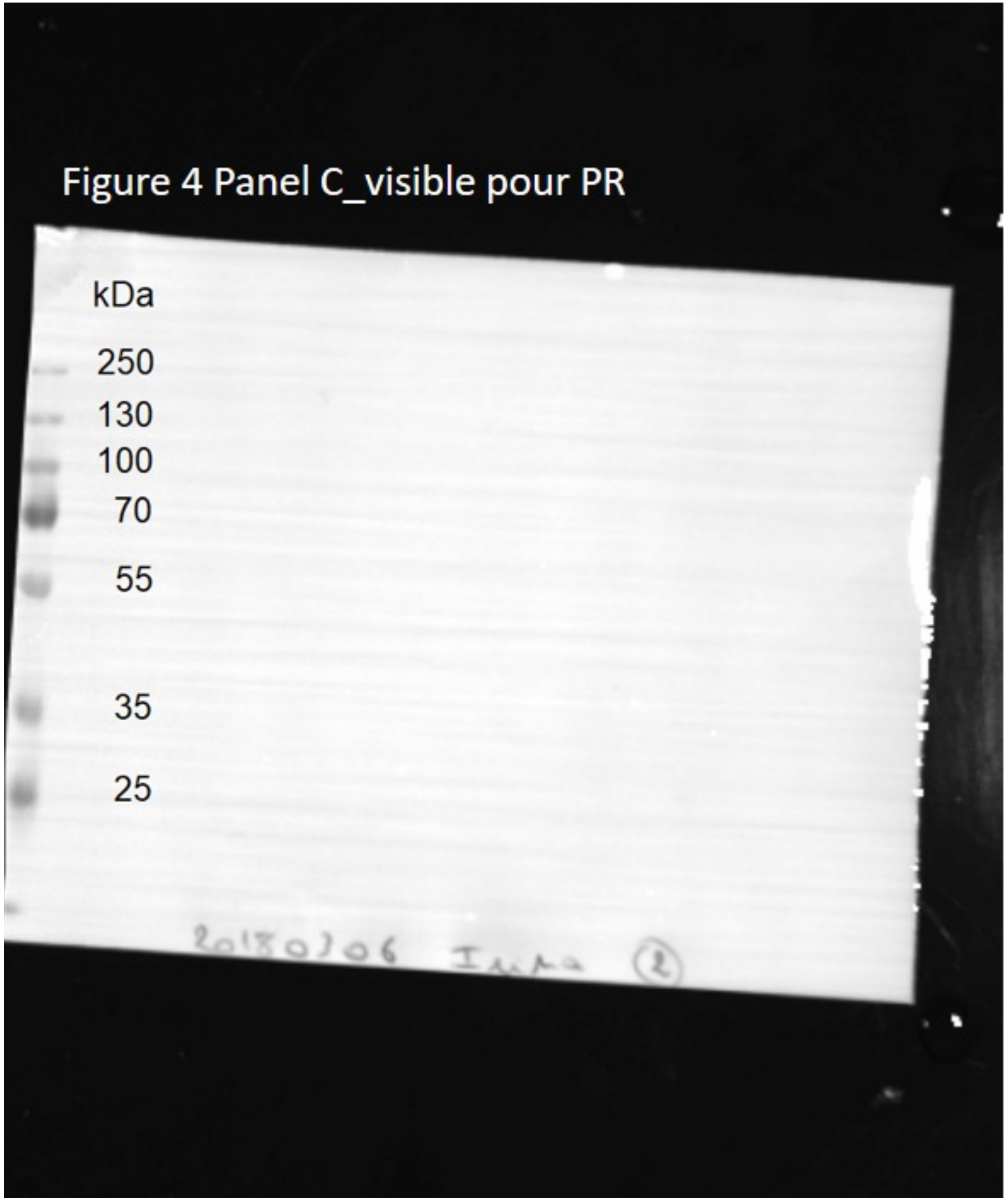
Figure 4 Panel C_visible pour PR
kDa
250
130
100
70
55
35
25

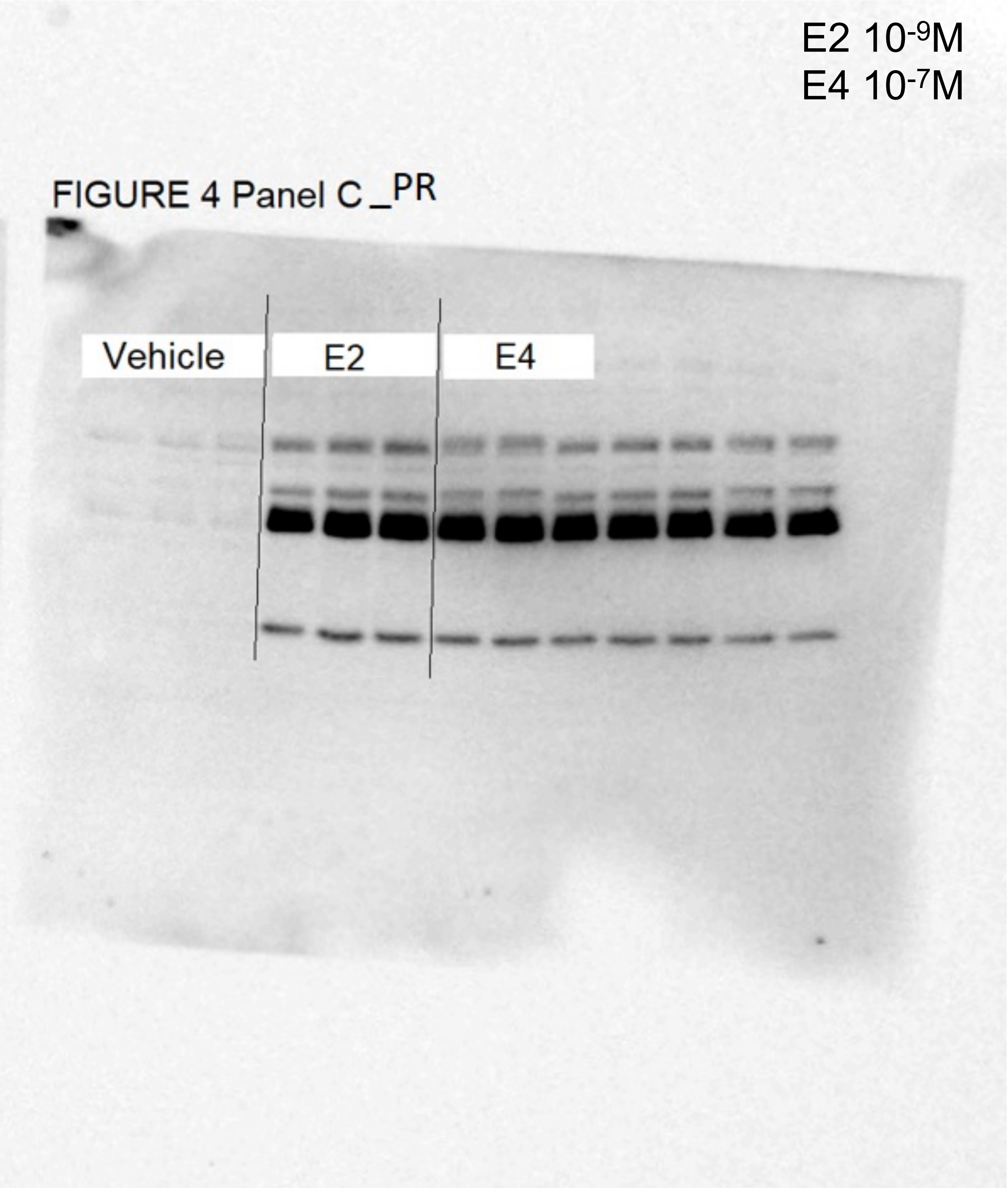
E2 10-9M
E4 10-7M
FIGURE 4 Panel C _PR
Vehicle
E2
E4

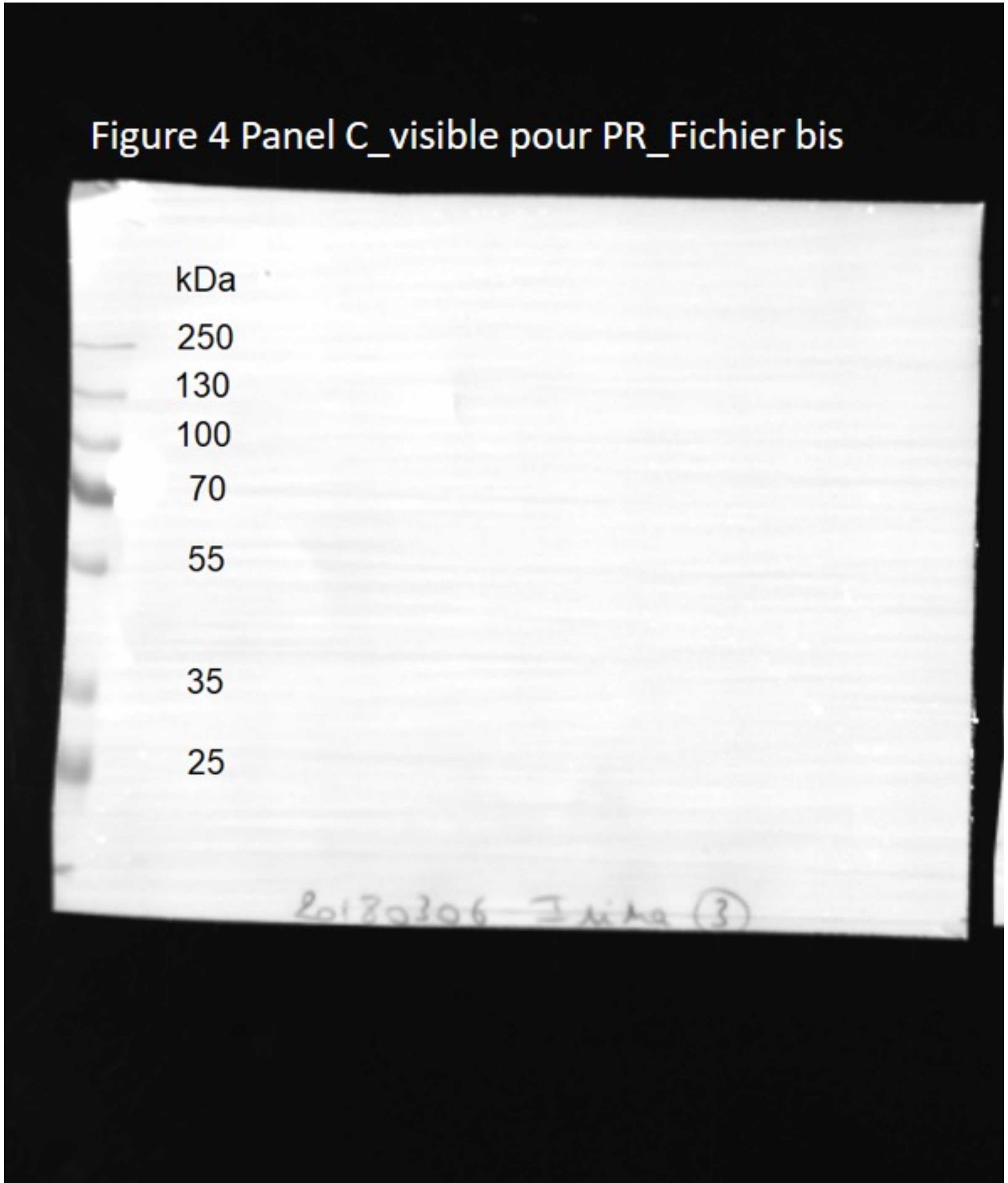
Figure 4 Panel C_visible pour PR_Fichier bis
kDa
250
130
100
70
55
35
25
20180306

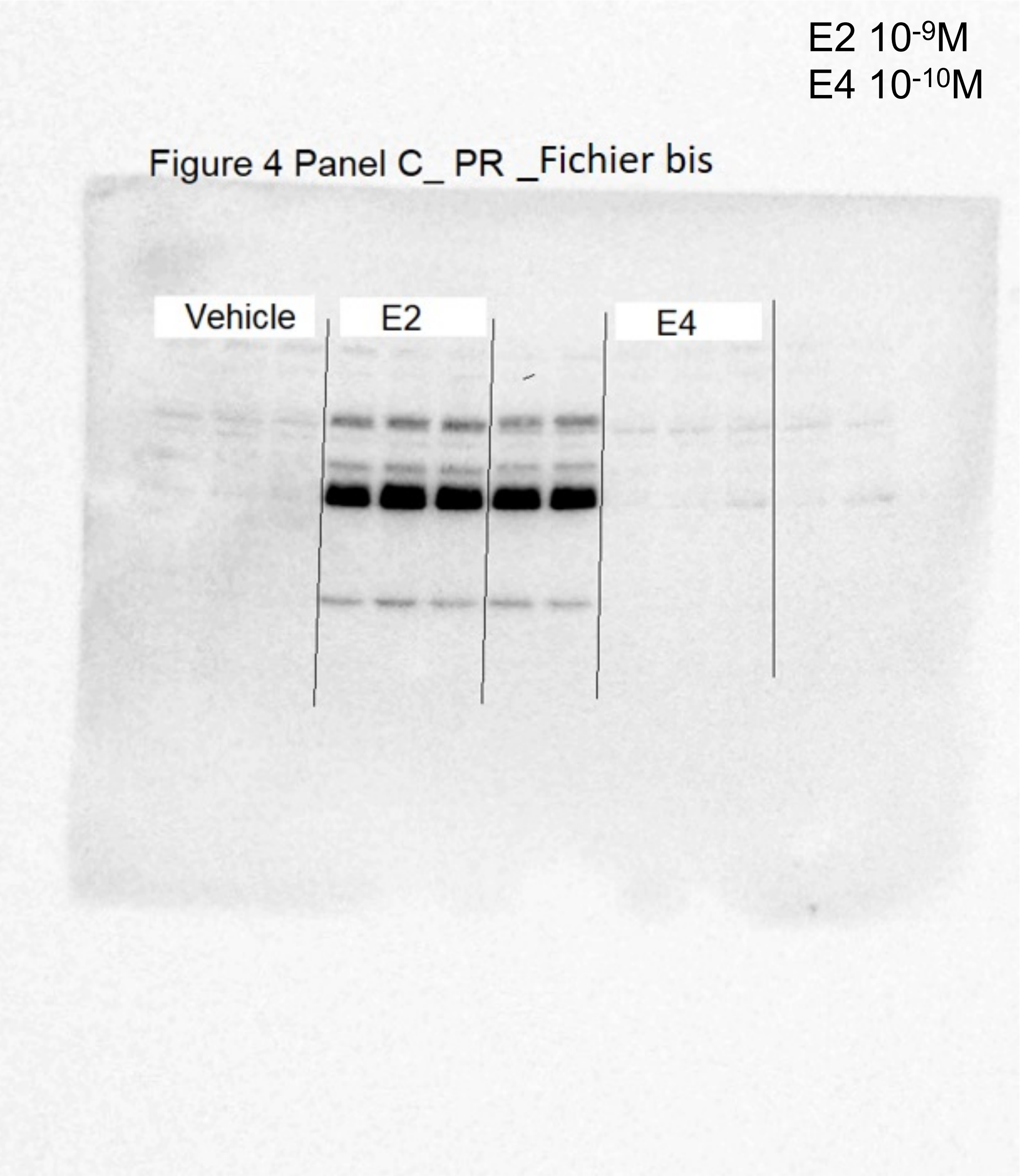
E2 10-9M
E4 10-10M
Figure 4 Panel C_ PR _Fichier bis
Vehicle
E2
E4

Figure 4 Panel C_visible pour ERalpha

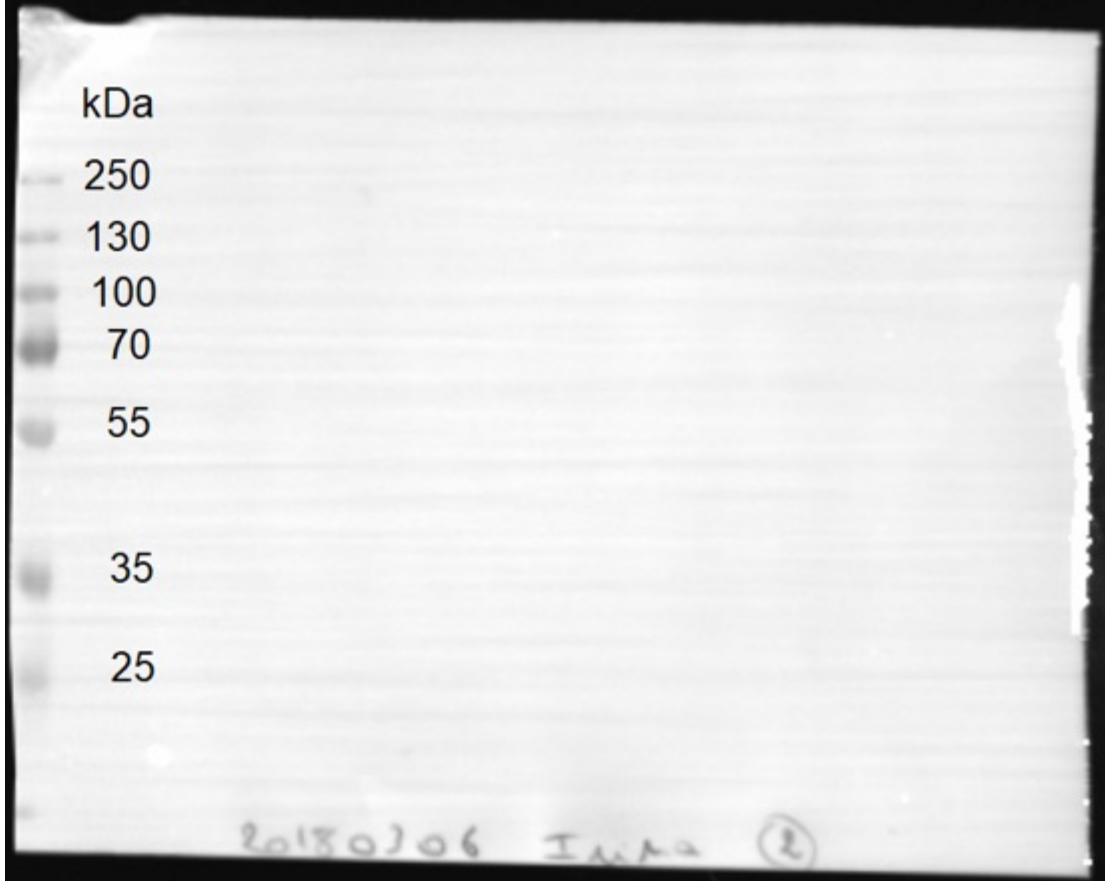

$E2\ 10^{-9}M$
$E4\ 10^{-7}M$

Figure 4 Panel C _ERalpha

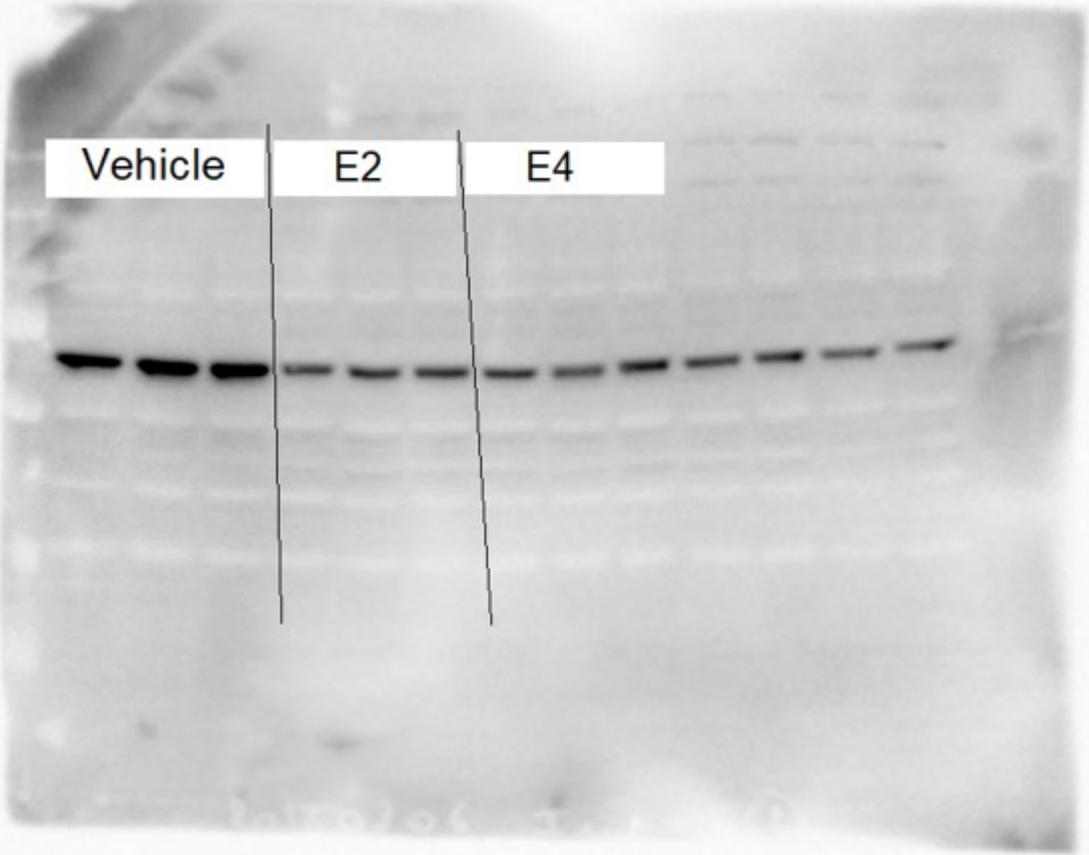

E2 10^{-9}M
E4 10^{-10}M

Figure 4 Panel C_visible pour Eralpha_fichier bis

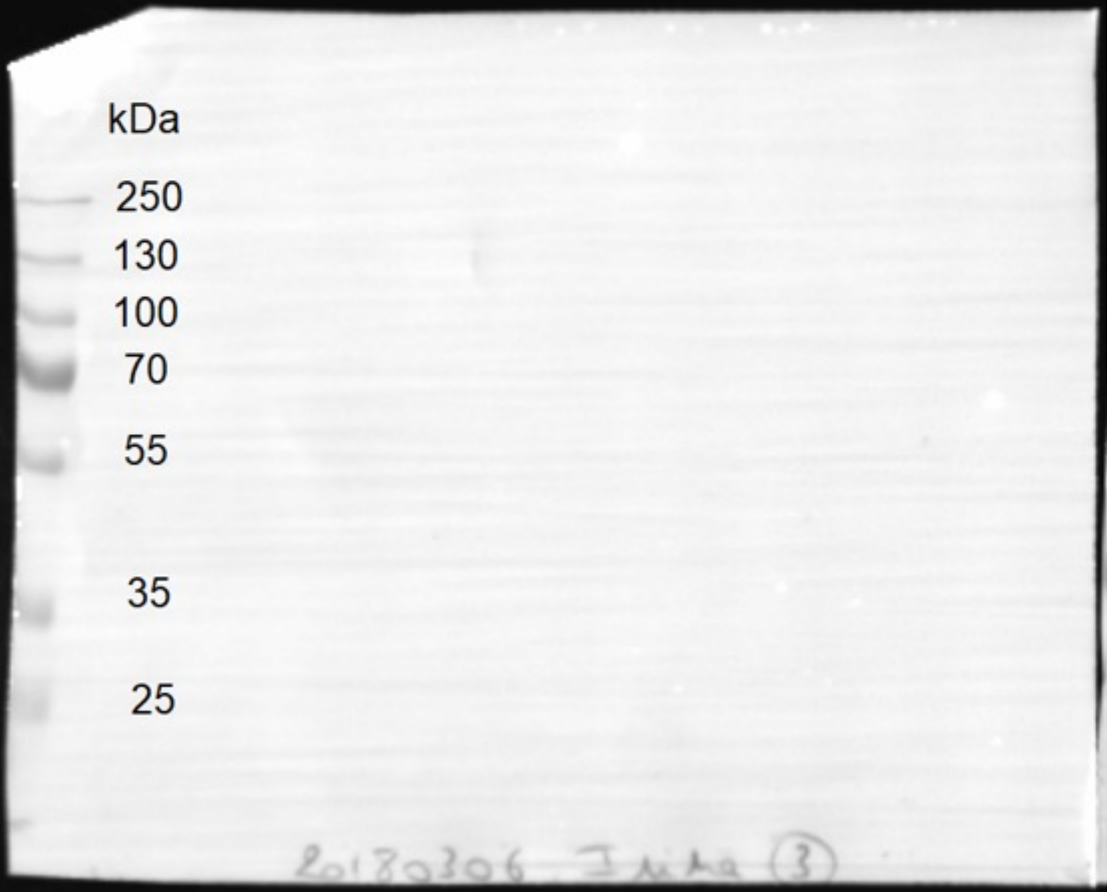

Figure 4 Panel C bis_ER _Fichier Bis

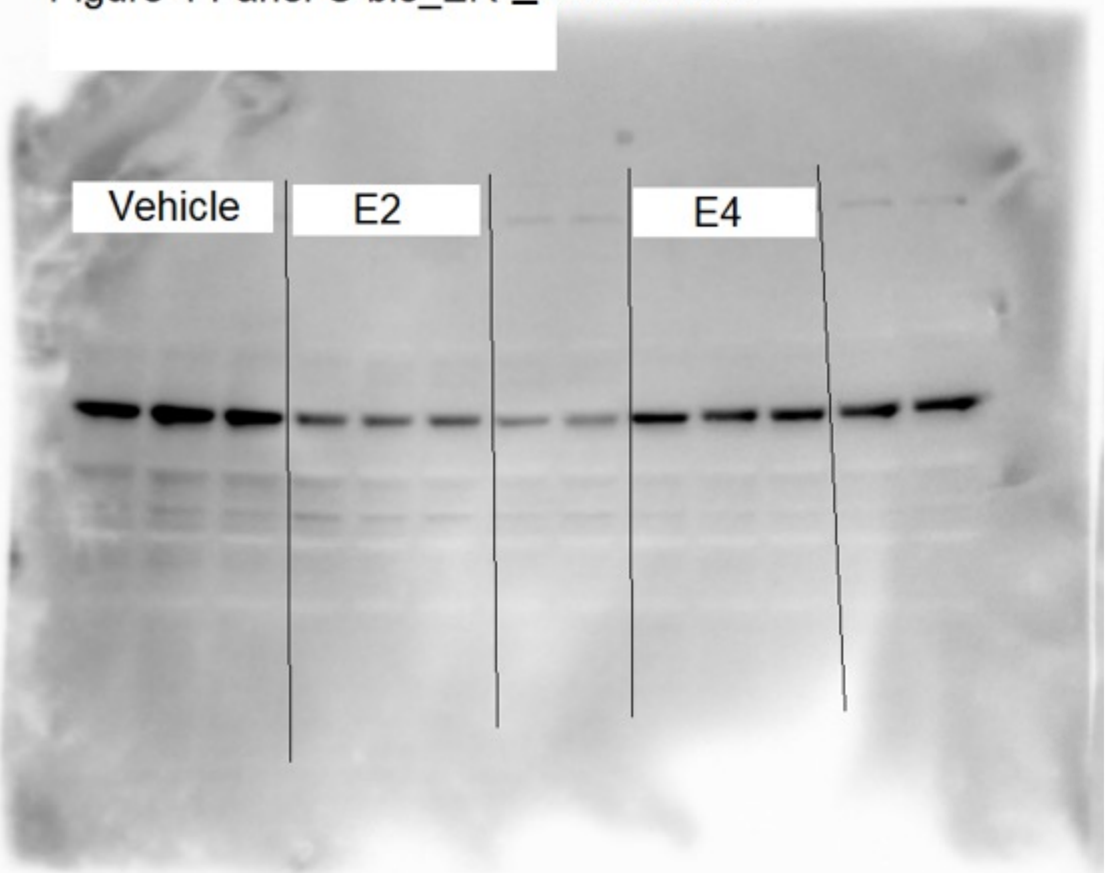

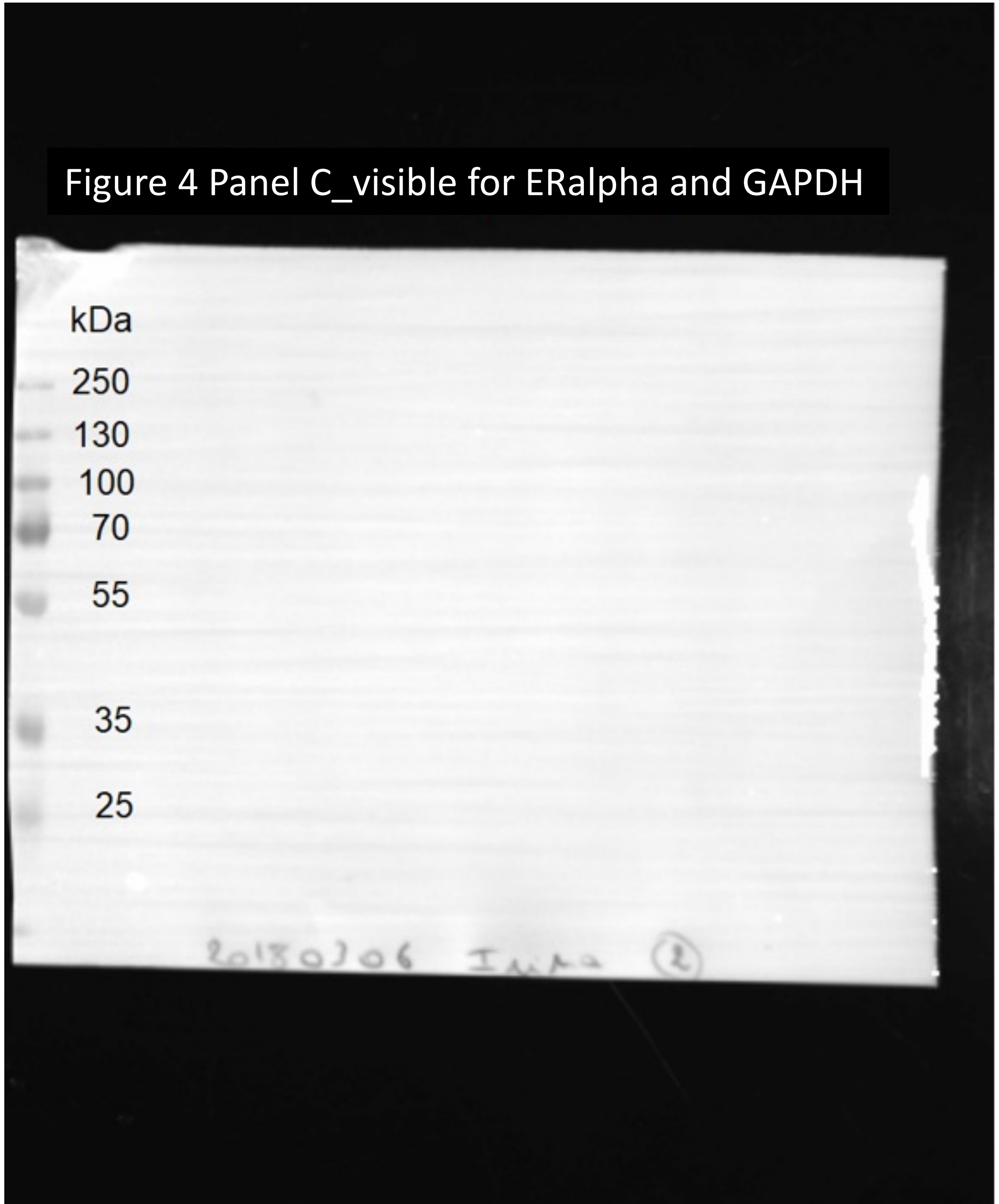
Figure 4 Panel C_visible for ERalpha and GAPDH
kDa
250
130
100
70
55
35
25

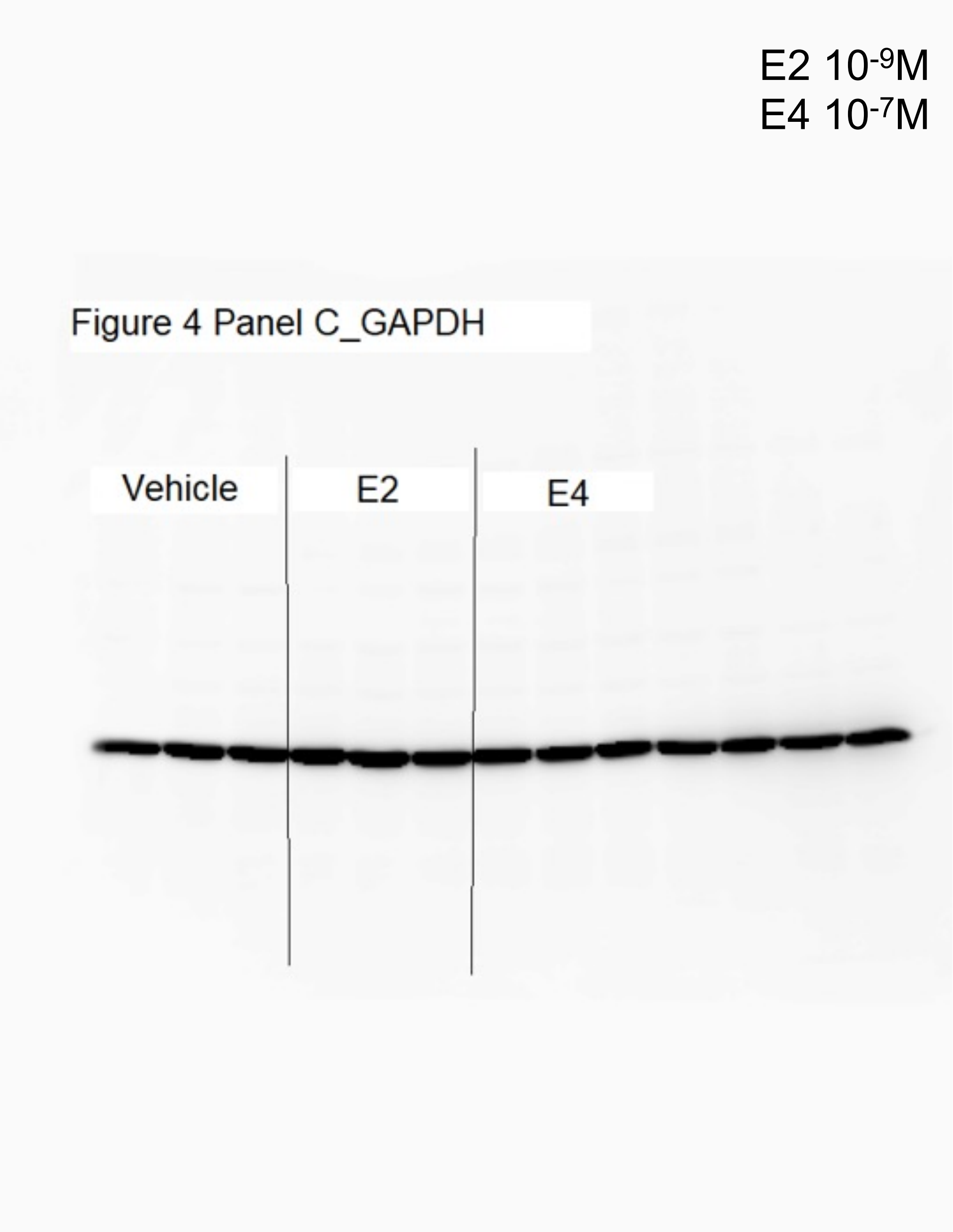
E2 10^{-9}M
E4 10^{-7}M
Figure 4 Panel C_GAPDH
Vehicle
E2
E4

$E2\ 10^{-9}M$
$E4\ 10^{-10}M$

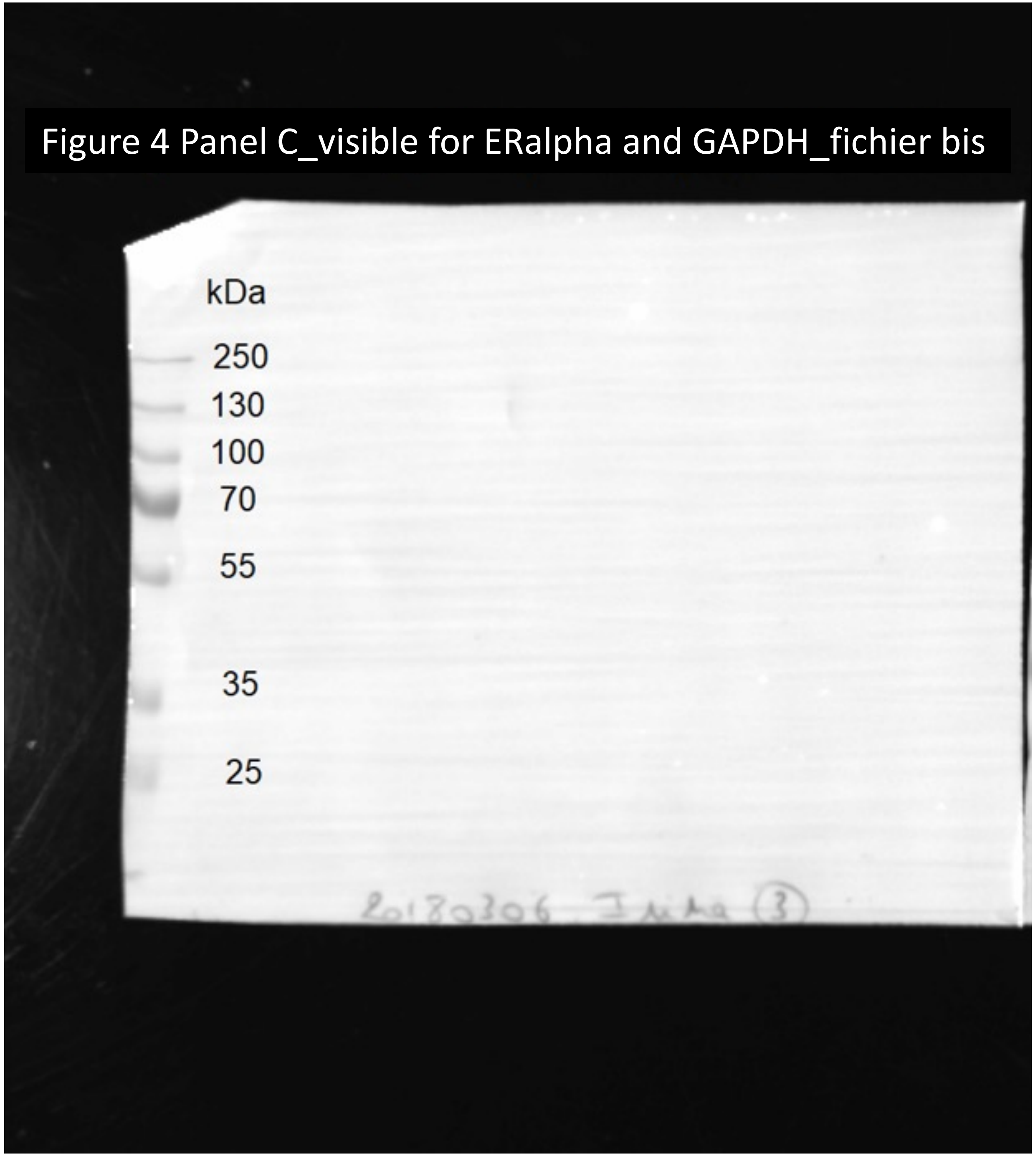

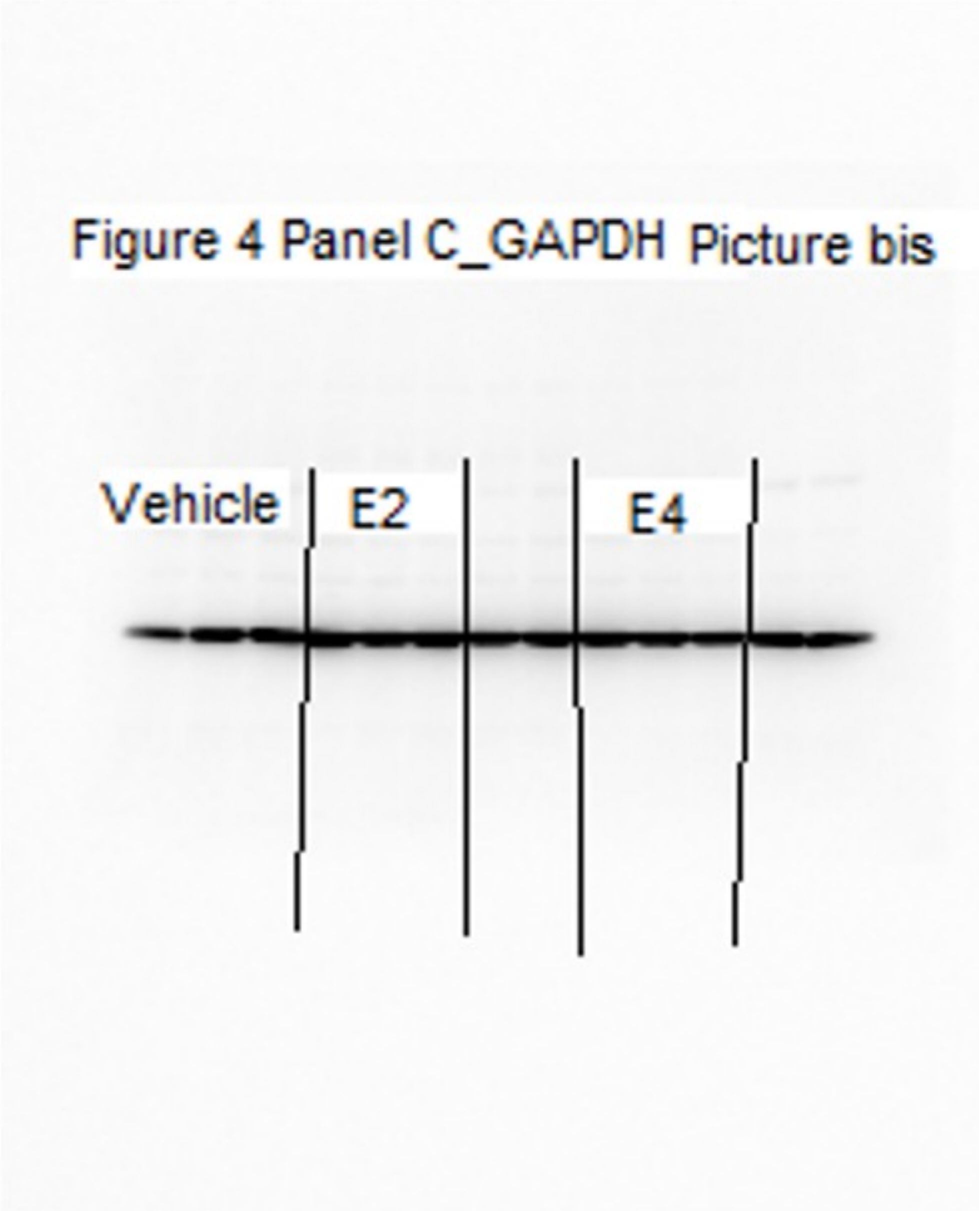

MCF7 Cells Western blot quantification

Figure 4 Panel C

PR/GAPDH Quantification			
**Vehicle**	**E2 10^{-9}M**	**E4 10^{-10}M**	**E4 10^{-7}M**
8,80728	16,55824	10,65471	22,86085
10,15728	20,24766	8,820607	22,89157
11,63038	22,81314	8,189587	22,2351
13,11502	18,28168		
9,208391	20,68641		
8,27153	20,65095		

n= 3-6 independent samples

Each sample was runned two times independently.
