# Supplementary material for: Estetrol Combined to Progestogen for Menopause or Contraception Indication Is Neutral on Breast Cancer
Source: Cancers (Basel). 2021 May 20;13(10):2486. doi: 10.3390/cancers13102486 (PMC8160902; doi:10.3390/cancers13102486)
Supplement: Supplementary file 1 [file cancers-13-02486-s001.zip › cancers-1178167_supplementary proof/Suppl Material_Fig 5 Panel H_WB whole blot.pdf]

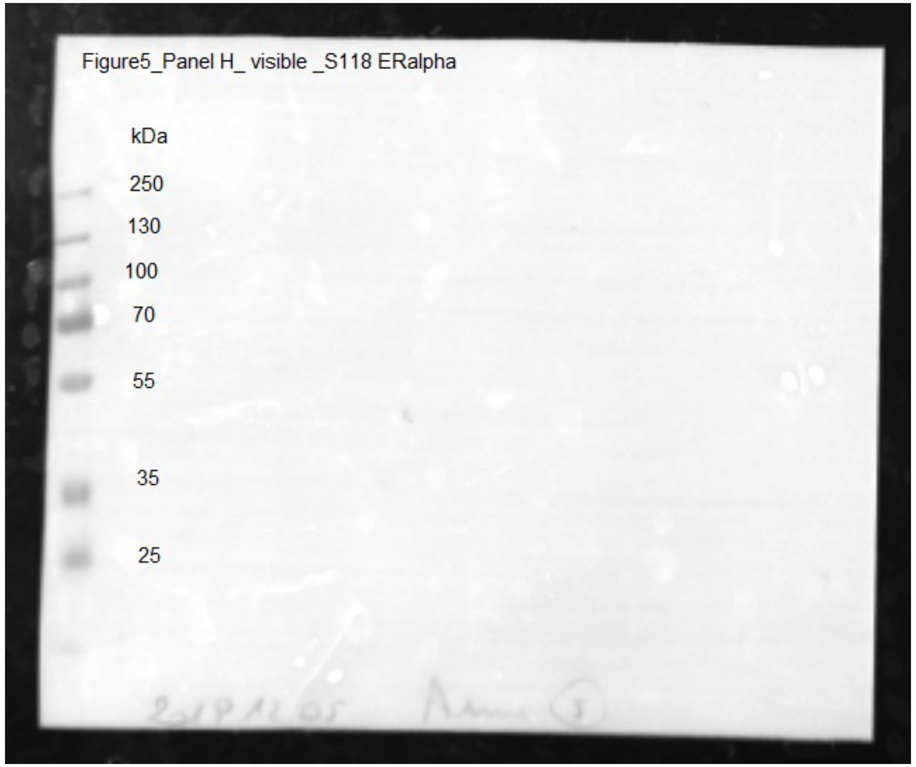

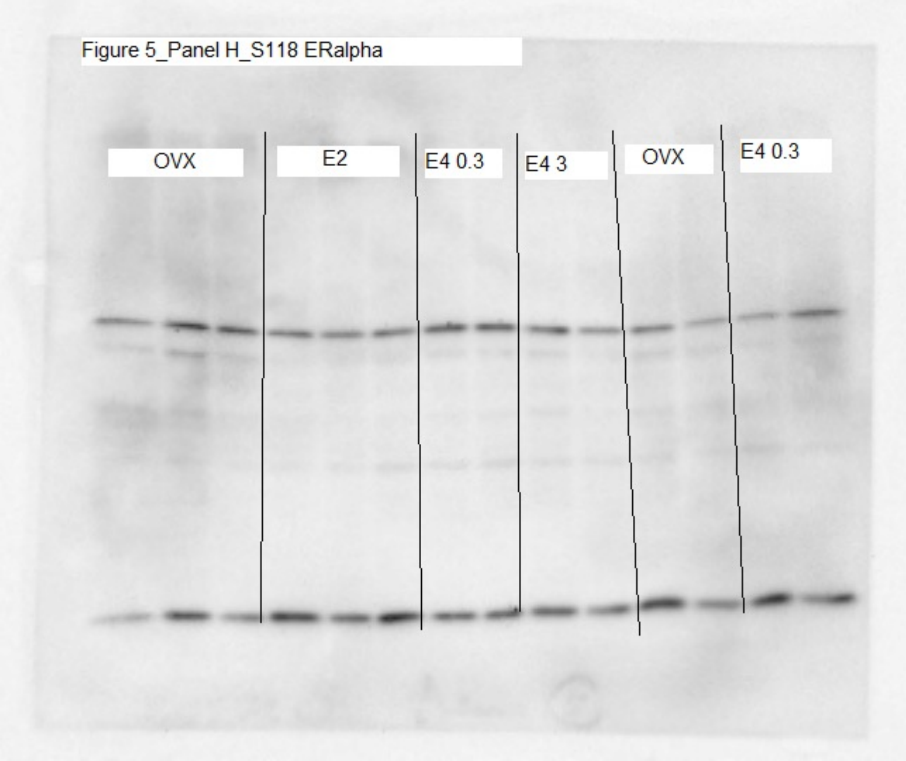

pS118-ERalpha 66kDa

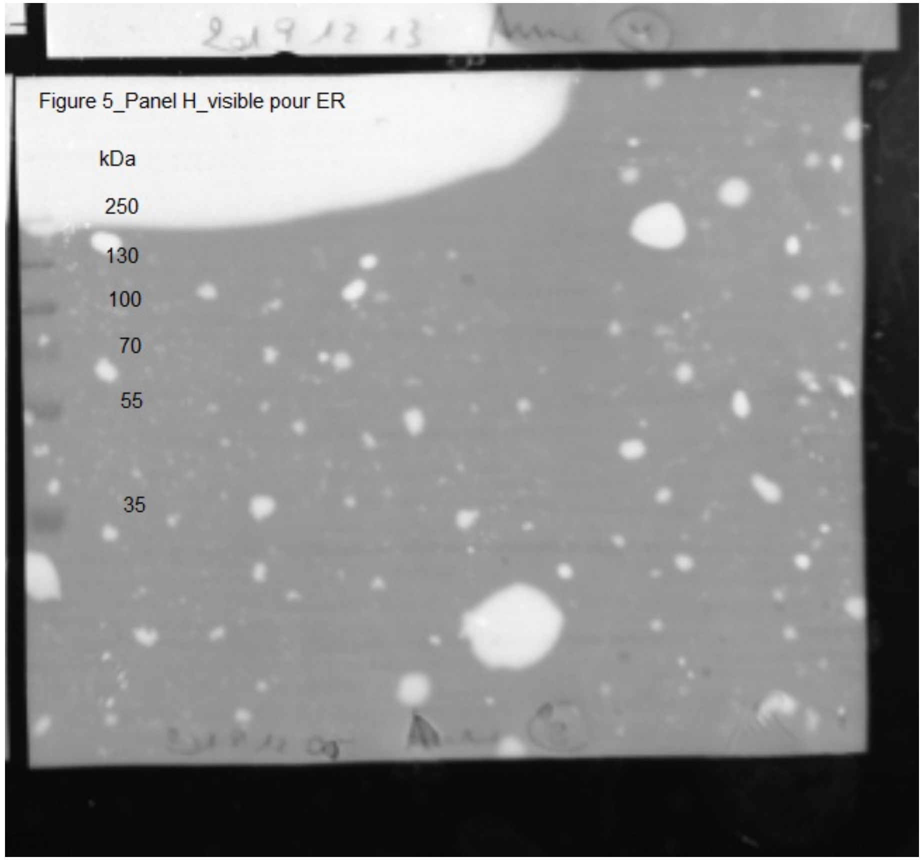

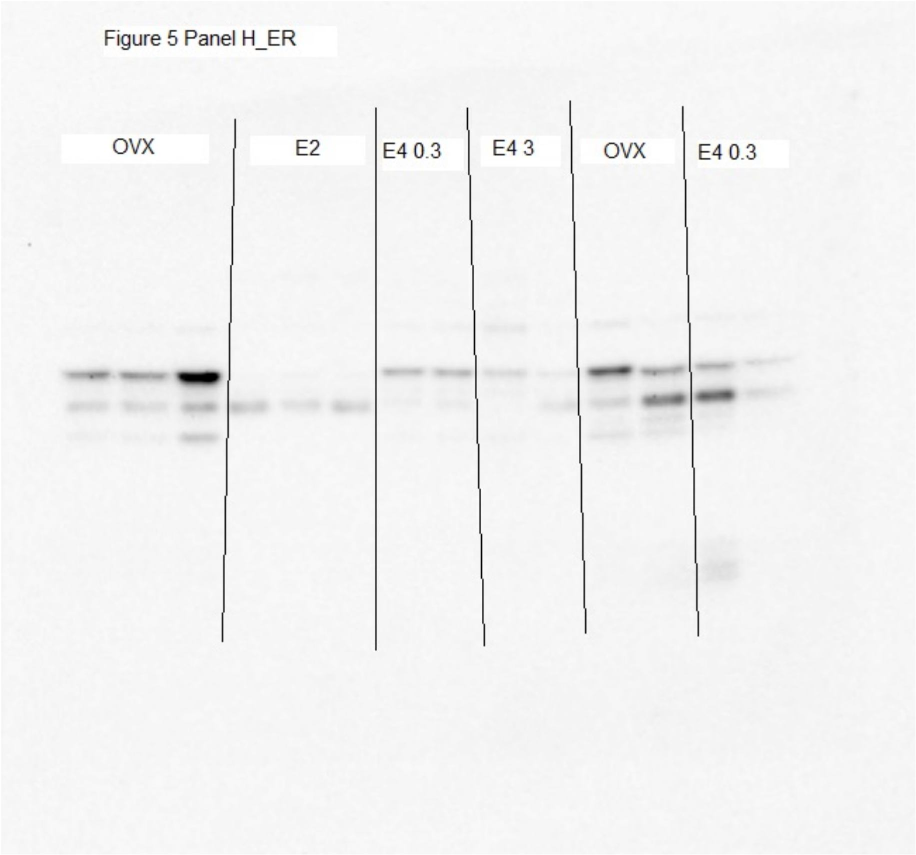

ERalpha 66kDa, 54kDa

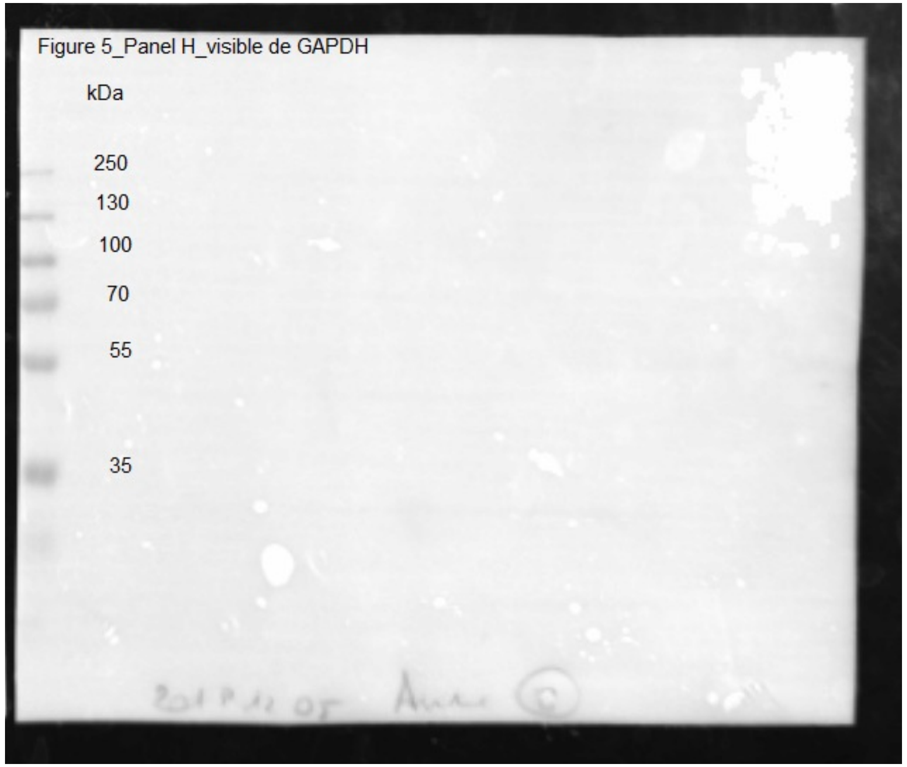

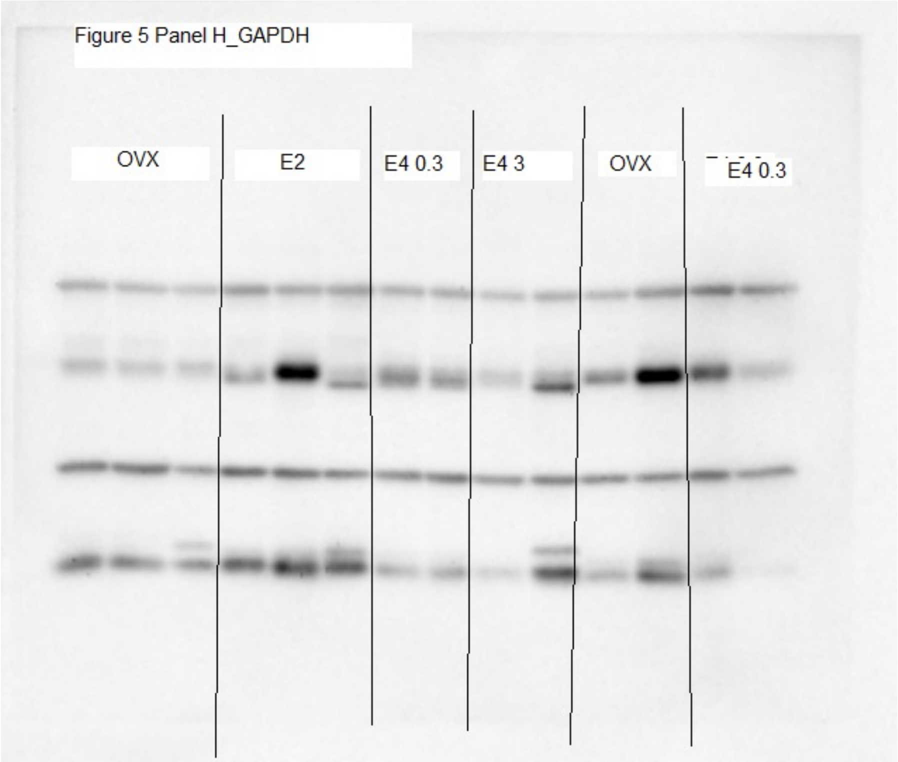

GAPDH 37kDa

Immunostainings have been made successively on the same membrane, using stripping between two immunostainings.

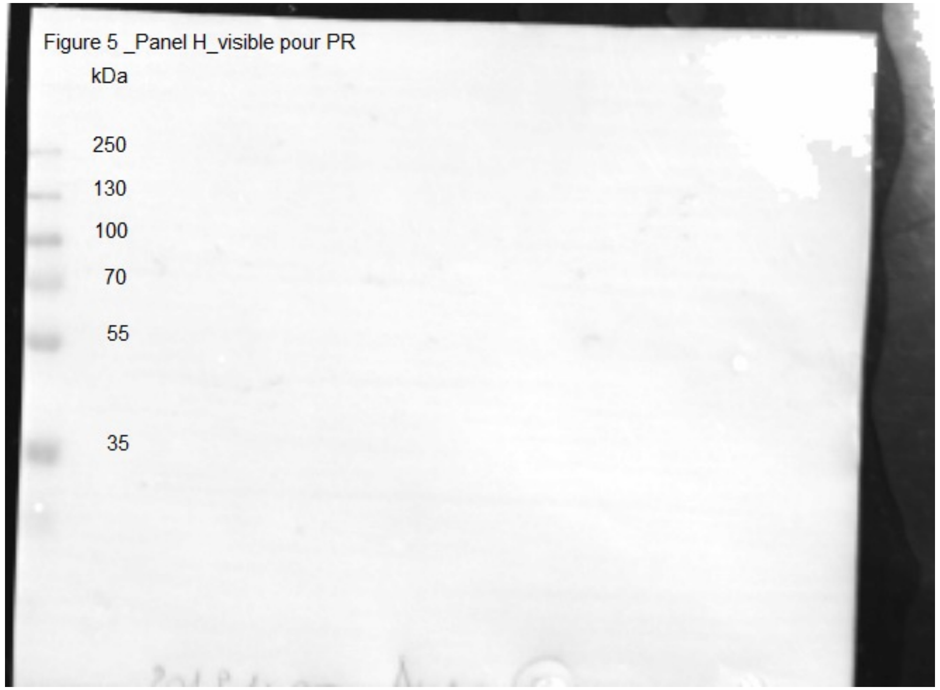

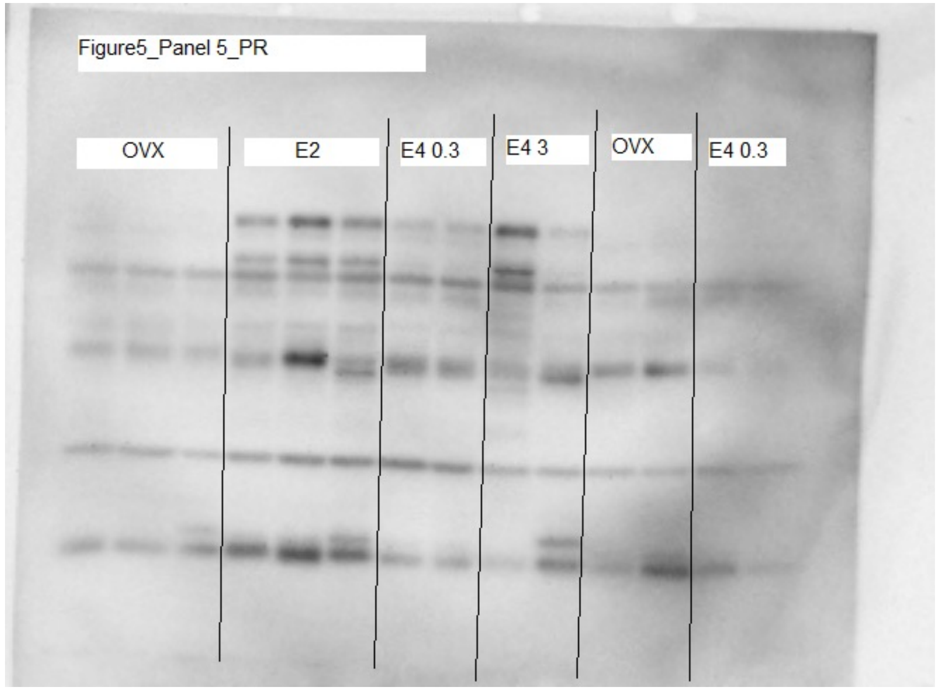

PRB 118kDa
PRA 90kDa

Immunostainings have been made successively on the same membrane, using stripping between two immunostainings.

PDX Tumors Western blot quantification					
Figure 5 Panel H					
PR/GAPDH Quantification					
W10				W35	
**OVX**	**E2**	**E4 0.3**	**E4 3**	**OVX**	**E4 0.3**
0,47998152	1,65968344	1,20369528	1,77831338	1,57022038	0,8746651
0,82287729	1,93847762	0,822899	1,70684831	0,67819482	0,77597595
0,86120565	1,92993962	0,61491538	1,22803994	0,52409644	0,66006152
0,3442122	1,67405663	0,66252814	1,57155689	0,5861421	0,90249824
0,35764802	0,68263502				
	0,82772675				

PDX Tumors Western blot quantification					
Figure 5 Panel H					
S118Eralpha/ERalpha Quantification					
W10				W35	
**OVX**	**E2**	**E4 0.3**	**E4 3**	**OVX**	**E4 0.3**
0,29740242	0,84790078	0,51536285	0,40745794	0,22944975	0,3101747
0,17212515	1,78500557	0,47590245	0,70294306	0,28487506	0,53875908
0,26585779	1,20311521	0,9777073	2,52952179	0,64354512	1,03314909
0,51945756	1,24800343	0,71373993	2,58603532	0,87584261	0,687056827
0,27575601	2,39352511				
	2,09065066				

n= 4-6 independant replicats
All the samples were separated in two gels.
Each sample was run two times independently.
